# Ovarian vein thrombosis mimicking acute abdomen: a case report and literature review

**DOI:** 10.1186/1749-7922-6-45

**Published:** 2011-12-23

**Authors:** Nikolaos Arkadopoulos, Dionysios Dellaportas, Anneza Yiallourou, Andreas Koureas, Dionysios Voros

**Affiliations:** 1 2nd Department of Surgery, Athens University School of Medicine, Aretaieion Hospital, Athens, Greece; 2 1st Department of Radiology, Athens University School of Medicine, Aretaieion Hospital, Athens, Greece

**Keywords:** ovarian vein, thrombosis, postpartum, appendicitis, appendectomy

## Abstract

**Background:**

Ovarian vein thrombosis (OVT) is a rare, but serious condition that affects mostly postpartum women. A high index of suspicion is required in order to diagnose this unusual cause of abdominal pain.

**Case presentation:**

A 19-year-old woman at three days postpartum was admitted to our hospital because of severe right lower quandrant abdominal pain and fever 38.5'C. Physical examination revealed an acutely ill patient and right lower quadrant tenderness with positive rebound and Giordano signs. The patient underwent appendectomy which proved to be negative for acute appendicitis. Postoperatively fever and pain persisted and abdominal CT-scan with intravenous contrast agent demonstrated a thrombosed right ovarian vein. The patient was initiated on low-molecular weight heparin (LMWH) and antibiotic treatment and a month later a new abdominal CT-scan showed a patent right ovarian vein.

**Discussion:**

Pathophysiologically, OVT is explained by Virchow's triad, because pregnancy is associated with a hypercoagulable state, venous stasis due to compression of the inferior vena cava by the uterus and endothelial trauma during delivery or from local inflammation. Common symptoms and signs of OVT include lower abdomen or flank pain, fever and leukocytosis usually within the first ten days after delivery. The reported incidence of OVT ranges 0,05-0,18% of pregnancies and in most cases the right ovarian vein is the one affected. Anticoagulation and antibiotics is the mainstay of treatment of OVT. Complications of OVT include sepsis, extension of the thrombus to the inferior vena cava and renal veins, and pulmonary embolism. The incidence of pulmonary embolism is reported to be 13.2% and represents the main source of mortality due to OVT.

**Conclusions:**

OVT is a rare condition, usually in the postpartum period. A high index of suspicion is required for the prompt diagnosis and management especially in cases that mimic acute abdomen.

## Background

Ovarian vein thrombosis (OVT) is a rare, but serious condition that affects mostly postpartum women but may also be associated with pelvic inflammatory disease, malignancies and pelvic surgical procedures. A high index of suspicion is required in order to diagnose this unusual cause of abdominal pain, which can mimic acute abdomen. A case of a 19-year-old at three days postpartum who suffered right lower quandrant pain and was operated for acute appendicitis, but turned out to suffer from OVT is presented herein, as well as modern diagnostic modalities and treatment options of this puzzling clinical condition. Also, a small review of the literature is attempted.

## Case presentation

**Figure 1:**
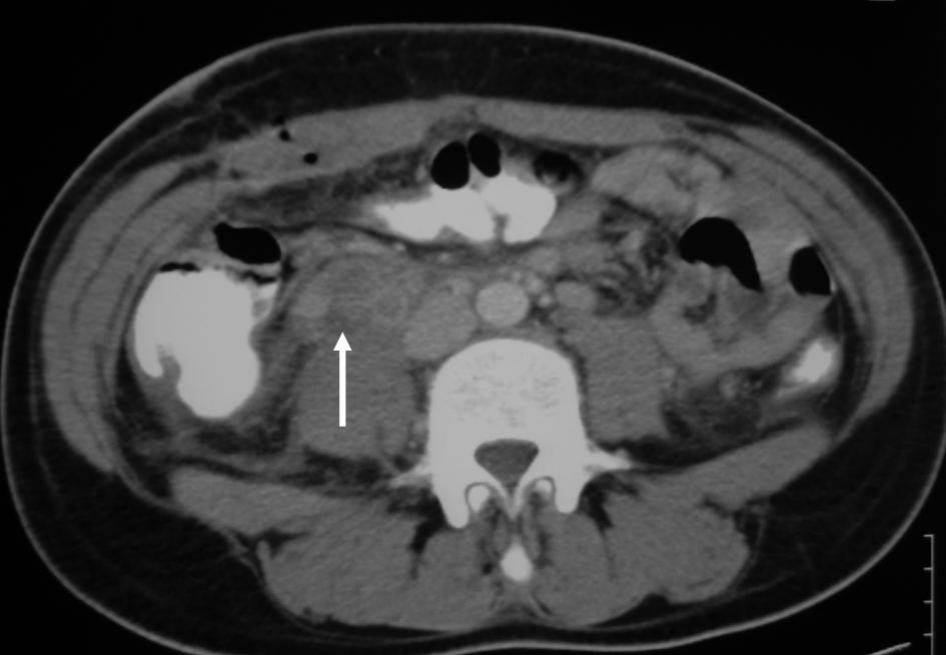
**Abdominal CT scan-arrow showing thrombosed right ovarian vein**.

**Figure 2:**
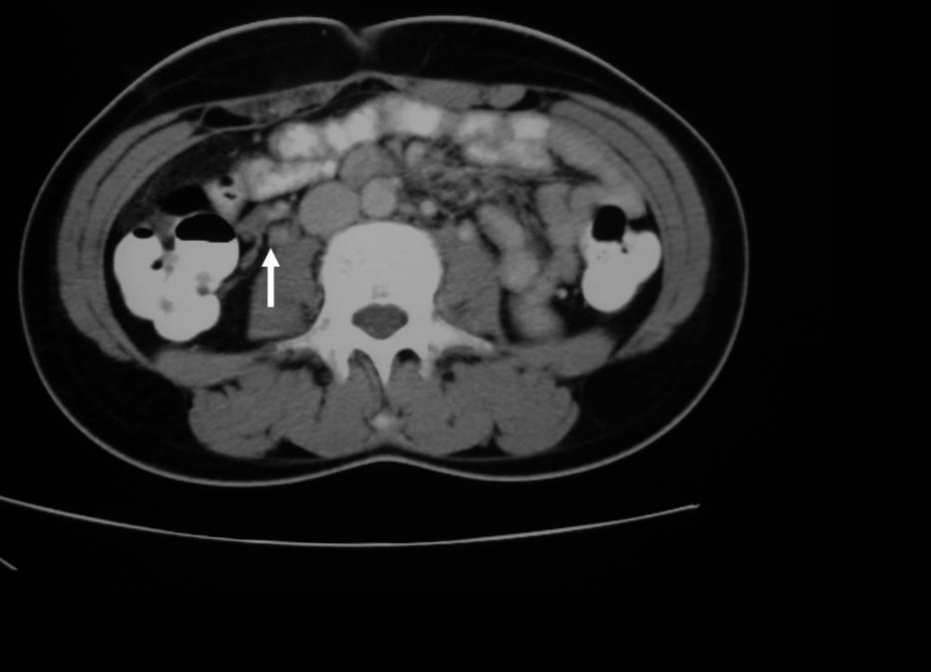
**Follow up abdominal CT scan one month after discharge-arrow indicating a patent right ovarian vein**.

A 19-year-old woman at three days postpartum was admitted to our hospital because of severe right lower quandrant abdominal pain. The pain started on postpartum day two and was accompanied with fever 38.5'C. There was no associated vaginal bleeding, but the patient complained of nausea and vomiting. She had vaginal delivery of a live born-term female, and the immediate postpartum period was uneventful. Physical examination showed an acutely ill patient. Heart rate was 110/min, blood pressure 110/75 mmHg and temperature was 38.3'C. Abdominal examination revealed right lower quadrant tenderness with positive rebound and Giordano signs. There was no evidence of deep vein thrombosis in the lower extremities. Laboratory exams revealed elevated white blood cell count (WBC 18500) with neutrophilia (89%) and elevated CRP (150 mg/dl). Abdominal and transvaginal ultrasound were unremarkable and the patient underwent appendectomy which proved to be negative for acute appendicitis. On the first postoperative day the patient's temperature was 38.4'C and a CT-scan with intravenous contrast agent was obtained. The latter revealed a thrombosed right ovarian vein (Figure 1) with stratification of the surrounding fat and signs of right ureteral dilatation. The patient was initiated on low-molecular weight heparin (LMWH) and antibiotic treatment with cefoxitin for five days. The patient was discharged on the 6^th ^postoperative day after switching LMWH to asenocoumarole. A month later the patient underwent a new abdominal CT-scan showing a patent right ovarian vein and improvement on the fat stratification (Figure 2). The patient is scheduled to discontinue asenocoumarole after three months of treatment and have laboratory examination for thrombofilia, as sometimes OVT is the first manifestation of such a condition [1].

## Discussion

The first case of postpartum ovarian vein thrombosis was described by Austin in 1956 [2]. Since then many authors have addressed this rare clinical condition. The 14 individual cases that have been reported so far are presented in Table 1. Pathophysiologically, OVT is explained by Virchow's triad, because pregnancy is associated with a hypercoagulable state, venous stasis due to compression of the inferior vena cava by the uterus and endothelial trauma during delivery or from local inflammation. The estimated incidence of OVT ranges between 0,05 and 0,18% of pregnancies with the majority of affecetd women being in the 3^rd ^or 4^th ^decade of their life. In 80-90% of the cases the right ovarian vein is the one affected, commonly 2-15 days following delivery. Cesarean delivery, also increases the risk of thrombosis to 1-2% and multiparity has been identified as a risk factor for thrombosis in general [3,4]. Rare causes of this entity are pelvic inflammatory disease, malignancies, Crohn's disease and pelvic surgical procedures [5,6]. Patients with malignant tumors, particularly those undergoing chemotherapy, are at risk for developing OVT, but is often asymptomatic and thrombus may resolve without any treatment [6]. Hypercoagulation conditions as systemic lupus erythematosus, antiphospholipid syndrome, presence of factor V Leiden, paroxysmal nocturnal haemoglobinuria, hyperhomocysteinaemia, protein C and S deficiency and heparin induced thrombocytopenia are all reported as risk factors for OVT [1,7].

**Table 1:** Individual case reports of ovarian vein thrombosis.

Authors	Risk factors	No of cases	Treatment	Surgical intervention
Austin OG [2]	Postpartum	1	Anticoagulation/antibiotics	No

Clarke CS et al [10]	Postpartum	1	Anticoagulation/antibiotics and IVC Greenfield filter	No

Sinha D et al [3]	Postpartum	1	Anticoagulation/antibiotics	No

Kominiarek MA et al [4]	Postpartum	1	Anticoagulation/antibiotics	Yes

Marcovici I et al [5]	Crohn's disease	1	Anticoagulation/antibiotics and Crohn's disease management	No

Jacoby WT et al [6]	Malignant tumor	6	Anticoagulation or observation	Νο

Tang LC et al [12]	Postpartum	1	Anticoagulation/antibiotics	Νο

Akinbiyi et al [13]	Postpartum	2	Anticoagulation/antibiotics	Νο

Royo P et al [14]	Postpartum	1	Anticoagulation/antibiotics	No

Common symptoms and signs of OVT include lower abdomen or flank pain, fever and leukocytosis usually within the first ten days after delivery [8]. A rare but characteristic coexistence is OVT with right ureteral obstruction and hydronephrosis, because anatomically the right ovarian vein crosses in front of the right ureter at the level of the L4 vertebra on its way to the inferior vena cava [8].

Diagnostic imaging can be performed using ultrasound, CT scan or MRI examinations, with magnetic resonance angiography having the best sensitivity and specifity. However the latter exam is reserved for doubtful situations and the two former are the most commonly used due to cost and speed considerations [9].

Diagnostic dilemma always occurs because of the rarity of this clinical entity. In cases when lower abdominal pain is the main symptom acute appendictitis cannot be excluded-leading to a negative appendectomy, as in our patient.

Anticoagulation and antibiotics is the mainstay of treatment of OVT. The morbidity of OVT arises from complcations such as sepsis, extension of the thrombus to the inferior vena cava and renal veins, and pulmonary embolism. The mortality of OVT can be as high as 5% and is mostly due to pulmonary embolism the incidence of which is reported to be 13.2% [10]. If the patient fails to respond to standard medical treatment or severe complications occur, options range from placement of an IVC Greenfield filter to hysterectomy and thrombectomy or even ligation of the inferior vena cava [11]. There are no recommendations for prophylaxis during a subsequent pregnancy, unless a hypercoagulable state is proved.

## Conclusions

OVT is a rare condition, usually in the postpartum period, with serious complications if left untreated. High index of suspicion is required for the prompt diagnosis and management especially in cases that mimic acute abdomen.

## Consent

Written informed consent was obtained from the patient for publication of this Case report and any accompanying images. A copy of the written consent is available for review by the Editor-in-Chief of this journal.

## List of abbreviations

CRP means C-reactive protein and CT-scan is used for computed tomography of the abdomen. The abbreviation OVT is for ovarian vein thrombosis. L4 is for fourth lumbar vertebrae and LMWH is used for low-molecular weight heparin. IVC indicates inferior vena cava and at least WBC is abbreviation for white blood cells.

## Competing interests

The authors declare that they have no competing interests.

## Authors' contributions

DD drafted the manuscript. AY analyzed the patient's clinical data and was major contributor in writing the manuscript, NA conceived and designed the study and and co-drafted the manuscript, AK analyzed the imaging studies. DV made substantial contributions to conception and design. All authors read and approved the final manuscript.
